# Supplementary material for: Effect of Precipitation Variation on Soil Respiration in Rain-Fed Winter Wheat Systems on the Loess Plateau, China
Source: Int J Environ Res Public Health. 2022 Jun 5;19(11):6915. doi: 10.3390/ijerph19116915 (PMC9180287; doi:10.3390/ijerph19116915)
Supplement: Supplementary file 1 [file ijerph-19-06915-s001.zip › ijerph-1738104-supplementary.pdf]

Table S1. Photosynthesis indexes of winter wheat in different phenological periods under precipitation variation

	Precipitation	Flowering stage	Milk ripening stage	Waxy ripe stage	Complete ripe stage
Photosynthetic rate (μmol m^{-2} s^{-1})	P_{-30}	18.48 b	11.94 a	13.11 b	7.45 b
	CK	19.86 b	15.02 a	17.10 a	16.31 a
	P_{+30}	25.36 a	16.41 a	18.11 a	16.40 a
Stomatal conductivity (mol m^{-2} s^{-1})	P_{-30}	0.25 b	0.11 a	0.13 b	0.06 b
	CK	0.25 b	0.13 a	0.18 a	0.17 a
	P_{+30}	0.44 a	0.16 a	0.22 a	0.17 a
Transpiration rate (mol m^{-2} s^{-1})	P_{-30}	0.0042 b	0.0020 b	0.0026 b	0.0017 b
	CK	0.0042 b	0.0023 b	0.0034 b	0.0042 a
	P_{+30}	0.0068 a	0.0029 b	0.0045 a	0.0040 a
Ntercellular CO_2 concentration (μmol m^{-2} s^{-1})	P_{-30}	244.79 b	198.84 a	208.45 b	191.8 b
	CK	236.59 b	184.44 a	221.57 ab	212.94 a
	P_{+30}	266.15 a	208.56 a	234.24 a	208.16 a

Different letters represent significant differences ($p < 0.05$) among treatments.

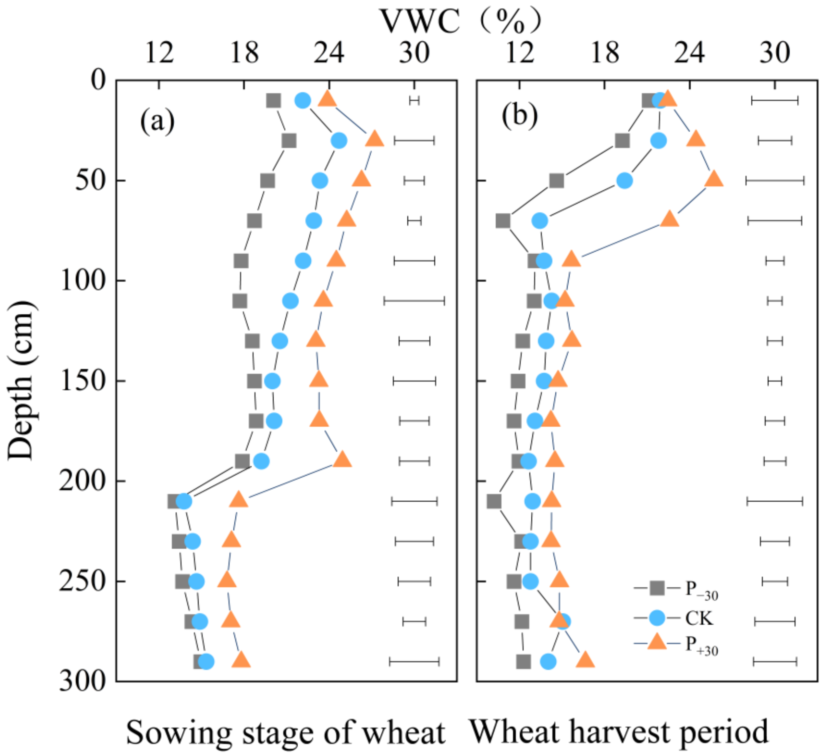

Figure S1. VWC of 0-300 cm in the soil profile during sowing and harvest of winter wheat, Horizontal bars represent LSD values based on the 0.05 significance level.
